# Micronized Vaginal Progesterone Dose and Serum Progesterone Thresholds Determine Reproductive Outcomes in Frozen–Thawed Embryo Transfer With Hormone Replacement Therapy

**DOI:** 10.1002/rmb2.70039

**Published:** 2026-03-12

**Authors:** Mami Sekiguchi, Ayumu Ito, Yukiko Katagiri, Masato Yoneyama, Takahiro Tsuchiya, Mayuko Furui, Nahomi Umemura, Hideyuki Kobayashi, Masahiko Nakata

**Affiliations:** ^1^ Department of Obstetrics and Gynecology, Faculty of Medicine Toho University Tokyo Japan; ^2^ Department of Obstetrics and Gynecology Toho University Omori Medical Center Tokyo Japan; ^3^ Reproduction Center Toho University Omori Medical Center Tokyo Japan; ^4^ Department of Urology Toho University Omori Medical Center Tokyo Japan

**Keywords:** artificial cycle, frozen–thawed embryo transfer, hormone replacement therapy, luteal phase support, micronized vaginal progesterone

## Abstract

**Purpose:**

To evaluate the combined effects of micronized vaginal progesterone (P4) dose and achieved serum P4 levels on reproductive outcomes in hormone replacement therapy–frozen embryo transfer (HRT‐FET) cycles by classifying patients according to both parameters.

**Methods:**

This retrospective cohort study analyzed 1137 single good‐quality blastocyst transfers performed between 2017 and 2022. Patients received OneCrinone 90 mg/day, Lutinus 300 mg/day, or Luteum 800 mg/day. Serum P4 levels on the day of embryo transfer (ET) and assisted reproductive technology (ART) outcomes were compared, including dose‐serum P4‐based subgroup analyses. Receiver operating characteristic analysis was used to explore a serum P4 cutoff for clinical pregnancy.

**Results:**

Higher daily doses were associated with increased serum P4 levels and higher implantation, clinical pregnancy, and live birth rates. An exploratory serum P4 cutoff of 13.1 ng/mL on the day of ET was associated with clinical pregnancy, with significantly better outcomes observed in patients with P4 ≥ 13.1 ng/mL. Dose‐serum P4‐based subgroup analyses showed that both parameters jointly influenced ART outcomes.

**Conclusion:**

In HRT‐FET cycles, achieving adequate micronized vaginal P4 dosage together with sufficient serum P4 levels on the ET day is associated with improved ART outcomes. Monitoring serum P4 may help support individualized luteal phase management.

## 1 Introduction

Recent advances in cryopreservation techniques have significantly improved the survival rates of embryos in assisted reproductive technology (ART) [1]. Controlled ovarian stimulation is used to obtain multiple oocytes, and a considerable number of embryos can be cryopreserved, thereby reducing the number of oocyte retrieval procedures. Moreover, freezing all embryos can prevent ovarian hyperstimulation syndrome [2]. Additionally, frozen–thawed embryo transfer (FET) reportedly results in higher pregnancy rates than fresh embryo transfer (ET) because of the possibility of better optimizing the endometrial hormonal environment for implantation [3]. Consequently, the importance of FET has recently increased, and its frequency has been increasing relative to that of fresh ET [4].

In HRT‐FET, unlike natural cycle frozen embryo transfer (NC‐FET), follicular development, ovulation, and corpus luteum formation are absent due to the negative feedback effects of exogenous hormone administration. Therefore, in HRT‐FET, in which corpus luteum formation is not observed, sufficient supplementation with progesterone (P4) is essential for the implantation and maintenance of pregnancy, unlike in fresh ET and NC‐FET, in which corpus luteum is present. Reportedly, the removal of the corpus luteum before the seventh to eightth week of pregnancy, when the main source of P4 shifts from the corpus luteum to the placenta, leads to a sudden drop in serum P4 levels, resulting in miscarriage [5]. P4 is mainly administered through oral, intramuscular, and vaginal routes. Among these, vaginal administration is considered particularly effective because the first uterine pass effect facilitates preferential delivery of P4 to the target organ, the endometrium [6]. Indeed, reports show that administering natural P4 via the vaginal route results in higher endometrial P4 concentrations, despite lower serum P4 levels than intramuscular administrations [7]. Owing to their low invasiveness and convenience of self‐administration, micronized vaginal P4 are widely used in hormone replacement therapy–frozen embryo transfer (HRT‐FET) cycles for luteal phase support.

However, it has been recently reported that in HRT‐FET, the use of micronized vaginal P4 is associated with significantly lower implantation, clinical pregnancy, and live birth rates, as well as significantly higher miscarriage rates than that of traditional intramuscular P4 [8]. Nevertheless, intramuscular P4 production has been discontinued in Japan, leaving only vaginal and oral formulations available for luteal phase support. Four types of micronized vaginal P4 are currently available in Japan, each with varying daily dosages of P4. While serum P4 levels may differ depending on the type of micronized vaginal P4 utilized in HRT‐FET [9], some studies have reported no significant impact on ART outcomes [10], whereas others suggest that higher daily P4 dosages lead to improved ART outcomes [11, 12]. Thus, no consensus currently exists regarding the optimal type or dose of micronized vaginal P4 for use in HRT‐FET. Hence, this study aimed to clarify the effects of currently available micronized vaginal progesterone doses and achieved serum progesterone levels on ART outcomes in HRT‐FET cycles, using a large single‐center Japanese real‐world cohort, by classifying patients according to both progesterone dose and serum progesterone levels.

## 2 Materials and Methods

### 2.1 Study Design and Patient Population

This retrospective cohort study was conducted at the Reproduction Center of Toho University Omori Medical Center. HRT‐FET cycles were included in which a single morphologically good‐quality blastocyst, without adjunctive treatments, was transferred between January 2017 and December 2022. Cycles were included in which one of three types of micronized vaginal P4 was utilized for luteal phase support: OneCrinone (Merck Biopharma, Tokyo, Japan), Lutinus (Ferring Pharmaceuticals, Saint‐Prex, Switzerland), or *Luteum* (ASKA Pharmaceutical, Tokyo, Japan). The daily dosages and administration frequencies followed the instructions provided in each medication's official package insert: OneCrinone 90 mg/day (once daily), Lutinus 300 mg/day (three times daily), and Luteum 800 mg/day (twice daily).

We excluded cycles involving multiple ETs, ETs with embryos that had undergone preimplantation genetic testing for aneuploidy, ETs based on endometrial receptivity analysis results, and cycles in which platelet‐rich plasma therapy was used as an adjunct treatment. The blastocysts were evaluated using the morphological scoring system defined by Gardner et al. [13, 14], and those graded as 3 BB or higher were classified as good‐quality blastocysts.

### 2.2 Protocol of Frozen–Thawed Blastocyst Transfer Cycle With Hormone Replacement Therapy and Outcome Definitions

HRT for FET was initiated on menstrual cycle day 3 using transdermal estradiol (E2) patches (Estrana TAPE 0.72 mg; Hisamitsu Pharmaceutical Co., Tokyo, Japan), administered every other day for about 10–14 days. The initial dosage was typically 1.44 mg, which was increased by 0.72 mg every 4 days, up to a maximum of 2.88 mg. In cases where the endometrial thickness had been insufficient or there was a history of follicular development during previous HRT, the E2 dose was appropriately increased, starting from 2.88 mg when necessary. After approximately 2 weeks of E2 administration, luteal phase support was initiated if the endometrial thickness reached at least 8.0 mm. The type of micronized vaginal P4 used was selected by the patient based on the information provided regarding formulation characteristics and dosing frequency. FET was performed on the fifth day after P4 administration. Serum P4 on the day of ET was measured at a fixed time; therefore, the blood draw corresponded to 6 h after administration of OneCrinone or Luteum and immediately after administration of Lutinus. Pregnancy was assessed by measuring serum human chorionic gonadotropin (hCG) levels 9 days after ET. Implantation was defined as a serum hCG level of ≥ 5 mIU/mL. E2 patches were continuously used until 7–8 weeks of gestation. Micronized vaginal P4 was continued until approximately 10 weeks of gestation. Clinical pregnancy was defined as the detection of a gestational sac within the uterus using transvaginal ultrasonography at 5 weeks of gestation. Ongoing pregnancy was defined as a pregnancy that continued beyond 12 weeks of gestation. A live birth was defined as the delivery of a live infant at or beyond 22 weeks of gestation. Miscarriage was defined as the loss of a clinically confirmed pregnancy before 22 weeks of gestation.

### 2.3 Outcomes

First, the groups were defined using micronized vaginal P4 as follows: the group using OneCrinone as P(90), Lutinus as P(300), and Luteum as P(800). Subsequently, we compared serum P4 levels on the day of ET, including the day of pregnancy testing. Implantation, clinical pregnancy, ongoing pregnancy, live birth, and miscarriage rates were also compared among these three groups.

Second, in the study population, we calculated a cutoff value for serum P4 levels on the day of ET that predicts clinical pregnancy. The proportion of cases that met this cutoff serum P4 level on the day of ET was compared among the three groups: P(90), P(300), and P(800). We compared the implantation, clinical pregnancy, ongoing pregnancy, live birth, and miscarriage rates between the groups above and below this cutoff value.

Third, the two groups P (90) and P (300)/P (800) were divided into four subgroups based on whether their serum P4 levels on the day of ET were below or above the cutoff value, and the implantation, clinical pregnancy, ongoing pregnancy, live birth, and miscarriage rates were compared for each group. The groups were labeled as follows: LL (< 300 mg, below the cutoff), LH (< 300 mg, above the cutoff), HL (≥ 300 mg, below the cutoff), and HH (≥ 300 mg, above the cutoff). We then compared the implantation, clinical pregnancy, ongoing pregnancy, live birth, and miscarriage rates among the four groups. In addition, logistic regression analysis was performed to evaluate ART outcomes using the Combined P4 dose–serum group (LL, LH, HL, HH), female age, body mass index (BMI), anti‐Müllerian hormone (AMH), baseline follicle‐stimulating hormone (FSH), baseline luteinizing hormone (LH), baseline E2, endometrial thickness on the day of ET scheduling, blastocyst expansion stage, inner cell mass grade, and trophectoderm grade as explanatory variables.

Implantation rate was defined as the number of implantations divided by the number of embryo transfers. Clinical pregnancy rate was defined as the number of clinical pregnancies divided by the number of embryo transfers. Ongoing pregnancy rate was defined as the number of ongoing pregnancies divided by the number of embryo transfers. Live birth rate was defined as the number of live births divided by the number of embryo transfers. Miscarriage rate was defined as the number of miscarriages divided by the number of clinical pregnancies.

### 2.4 Statistical Analyses

All statistical analyses were performed using SPSS version 29 (IBM Corp., Armonk, NY, USA). Continuous variables were first tested for normality. For between‐group comparisons, analysis of variance was used for normally distributed variables, and the Kruskal–Wallis test was used for non‐normally distributed variables. Post hoc analyses were performed as appropriate. Categorical variables were compared among groups using the chi‐squared test or Fisher's exact test. The predictive value of serum P4 level on the day of ET for clinical pregnancy was assessed using receiver operating characteristic (ROC) curve analysis, with the area under the curve (AUC), sensitivity, specificity, and the Youden index calculated. The cutoff value of serum P4 was determined by the maximum Youden index. A *p* < 0.05 was considered statistically significant.

## 3 Results

### 3.1 Patient Characteristics

Overall, 1137 HRT‐FET cycles for 596 patients were included in the analysis. The number of cases by the number of ET attempts was 293 for one, 166 for two, 82 for three, 28 for four, and 27 for five or more. Of these, 520, 202, and 415 cycles were performed in the P(90), P(300), and P(800) groups, respectively. In addition, the calendar‐year distribution of vaginal progesterone formulations (P(90), P(300), and P(800)) during the study period is presented in Figure S1. The patient characteristics are summarized in Table 1. The median age was 37, 39, and 37 years in the P(90), P(300), and P(800) groups, respectively. Significant differences were observed between the P(90) and P(300) groups and between the P(300) and P(800) groups (both *p* < 0.01). The mean BMI was 22.2 ± 3.8, 23.1 ± 4.2, and 22.3 ± 3.5 kg/m^2^ in the P(90), P(300), and P(800) groups, respectively, with a significant difference between the P(90) and P(300) groups (*p* < 0.01). No significant difference was observed between the P(300) and P(800) groups. Baseline E2 levels were significantly lower in the P(300) group (33.2 ± 22.3 pg/mL) than in the P(90) (39.5 ± 24.7 pg/mL) and P(800) (36.2 ± 23.6 pg/mL) groups (*p* = 0.005). The mean serum P4 levels on the day of ET were 11.7 ± 4.7, 14.1 ± 7.2, and 14.8 ± 5.8 ng/mL in the P(90), P(300), and P(800) groups, respectively (both *p* < 0.01). On the pregnancy test day, the mean serum P4 levels were 10.4 ± 9.8, 12.8 ± 5.5, and 15.8 ± 9.9 ng/mL for the P(90), P(300), and P(800) groups, respectively (all *p* < 0.01). No significant differences were observed in AMH levels, endometrial thickness on the day of ET scheduling, or baseline FSH and LH levels among the groups.

**TABLE 1:** Patient characteristics.

	P(90) *n* = 520	P(300) *n* = 202	P(800) *n* = 415	*p*
Female age (years)	37 (24–48)^a^	39 (25–45)^b^	37 (22–45)^a^	< 0.001
BMI (㎏/㎡)	22.2 ± 3.8^a^	23.1 ± 4.2^b^	22.3 ± 3.5^a,b^	0.04
AMH (ng/mL)	3.2 ± 2.7	3.3 ± 3.1	3.3 ± 2.8	0.989
Baseline FSH (mIU/mL)	7.6 ± 3.1	7.5 ± 3.4	7.5 ± 3.3	0.977
Baseline LH (mIU/mL)	5.1 ± 3.2	4.7 ± 2.5	4.7 ± 2.4	0.333
Baseline E2 (pg/mL)	39.5 ± 24.7^a^	33.2 ± 22.3^b^	36.2 ± 23.6^a^	0.005
Endometrial Thickness on ET Scheduling Day (mm)	9.9 (6.4–17.3)	9.8 (6.6–19.0)	10.0 (6.2–18.7)	0.296
Serum E2 level on ET Day (pg/mL)	292 ± 183	269 ± 171	281 ± 179	0.085
Serum P4 level on ET Day (ng/mL)	11.7 ± 4.7^a^	14.1 ± 7.2^b^	14.8 ± 5.8^b^	< 0.001
Serum P4 level on Pregnancy test Day (ng/mL)	10.4 ± 9.8^a^	12.8 ± 5.5^b^	15.8 ± 9.9^b^	< 0.01

*Note:* For continuous variables, data are expressed as the median (range: minimum–maximum) or as the mean ± standard deviation. Superscript letters indicate statistical comparison: values sharing the same letter are not significantly different, whereas values with different letters differ significantly.

Abbreviations: AMH, anti‐Müllerian hormone; BMI, body mass index; ET, embryo transfer; FSH, follicle‐stimulating hormone; LH, luteinizing hormone; P4, progesterone.

### 3.2 Outcomes

**FIGURE 1:**
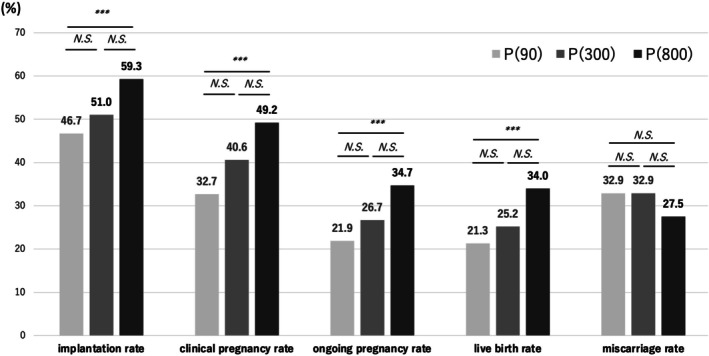
The reproductive outcomes in the P(90), P(300), and P(800) groups. *n* = 1,137 cycles (P(90): 520; P(300): 202; P(800): 415). ART, assisted reproductive technology; P(90), the group using Crinone; P(300), the group using Lutinus; P(800), the group using Luteum. ***p < 0.001; N.S., not significant.

In the P(90), P(300), and P(800) groups, the ART outcomes were as follows: implantation rates were 46.7% (243/520), 51.0% (103/202), and 59.3% (246/415); clinical pregnancy rates were 32.7% (170/520), 40.6% (82/202), and 49.2% (204/415); ongoing pregnancy rates were 21.9% (114/520), 26.7% (54/202), and 34.7% (144/415); and live birth rates were 21.3% (111/520), 25.2% (51/202), and 34.0% (141/415), respectively. These outcomes showed a sequential increasing trend in accordance with the daily dose of micronized vaginal P4 (Figure 1). All four ART outcomes showed significant differences between the P(90) and P(800) groups (all *p* < 0.01). Nonetheless, miscarriage rates were 32.9% (56/170), 32.9% (27/82), and 27.5% (56/204), in the P(90), P(300), and P(800) groups, respectively. These rates did not significantly differ among the three groups.

The cutoff serum P4 level on the day of ET for clinical pregnancy (ROC‐AUC [95% confidence interval]) was 13.1 ng/mL (0.54 [0.51–0.58]).

**FIGURE 2:**
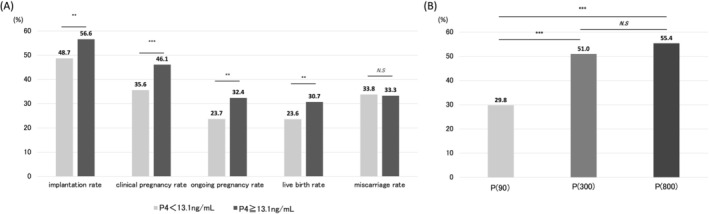
(A) The reproductive outcomes for groups with P4 levels equal to or above or below the cut‐off value. (B) The proportions of patients meeting the cutoff value (serum progesterone (P4) level on the day of embryo transfer for clinical pregnancy was 13.1 ng/mL) in the P(90), P(300), and P(800) groups. *n* = 1137 cycles (P(90): 520; P(300): 202; P(800): 415). Serum P4 on the day of ET: < 13.1 ng/mL, *n* = 649; ≥ 13.1 ng/mL, *n* = 488. P(90), the group using Crinone; P(300), the group using Lutinus; P(800), the group using Luteum; ET, embryo transfer. P4, progesterone; ART, assisted reproductive technology; ET, embryo transfer. ***p < 0.001; N.S, not significant.

The study population was then divided into two groups based on this cutoff value: those with serum P4 levels at or above the cutoff and those with levels below the cutoff. The implantation, clinical pregnancy, ongoing pregnancy, and live birth rates were all significantly higher in the group with P4 levels at or above the cutoff than in those below the cutoff (all *p* < 0.01). The miscarriage rates did not significantly differ between the two groups (Figure 2A).

The proportions of patients meeting the cutoff values in the P(90), P(300), and P(800) groups were 29.8% (155/520), 51.0% (103/202), and 55.4% (230/415), respectively. Significant differences were observed between P(90) and P(300), and between P(90) and P(800) (all *p* < 0.01) (Figure 2B).

**FIGURE 3:**
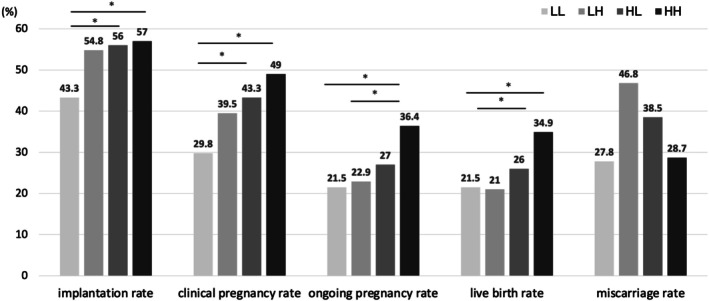
The reproductive outcomes were compared among four groups classified according to the daily dose of micronized vaginal progesterone and the serum progesterone level on the day of embryo transfer. Combined P4 dose–serum P4 groups: LL, *n* = 363; LH, *n* = 157; HL, *n* = 282; HH, *n* = 335. LL, low dose P4 (using Crinone) and below cut‐off value; LH, low dose P4 (using Crinone) and equal to or above cut‐off value; HL, high dose P4 (using Lutinus and Luteum) and below cut‐off value; HH, high dose P4 (using Lutinus and Luteum) equal to or above cut‐off value; ART, assisted reproductive technology; P4, progesterone ET, embryo transfer. *p < 0.05; N.S., not significant.

In the comparison based on the four groups defined by the P4 daily dose and serum P4 level on the day of ET, the patient characteristics of the LL, LH, HL, and HH groups are summarized in (Table S1). Female age differed significantly among the groups (*p* = 0.002), with the LL group showing a slightly younger median age than the other groups. BMI also differed modestly among the groups (*p* = 0.03). Baseline FSH, baseline LH, and baseline E2 levels differed significantly among the groups (all *p* < 0.001). In contrast, AMH levels and endometrial thickness on the ET scheduling day did not differ significantly among the groups (*p* = 0.354 and *p* = 0.11, respectively). Implantation, clinical pregnancy, ongoing pregnancy, and live birth rates increased progressively across the LL, LH, HL, and HH groups. No significant differences in miscarriage rates were observed among the four groups. For implantation and clinical pregnancy rates, the LL group showed significant differences compared with the HL and HH groups (all *p* < 0.05). For ongoing pregnancy and live birth rates, the HH group showed significant differences compared with the LL and LH groups (all *p* < 0.05) (Figure 3). Logistic regression analysis further revealed that female age was a statistically significant negative predictor of ART outcomes (Table 2). In contrast, the Combined P4 dose–serum group was identified as a statistically significant positive predictor for implantation, clinical pregnancy, ongoing pregnancy, and live birth. The adjusted odds ratios (95% confidence intervals) were 1.266 (1.117–1.434), 1.412 (1.241–1.608), 1.393 (1.209–1.605), and 1.349 (1.171–1.555), respectively.

**TABLE 2:** Multivariable logistic regression analysis of factors associated with ART outcomes.

Predictor	Adjusted odds ratio for ART outcomes (95% CI)				
	Implantation	Clinical pregnancy	Ongoing pregnancy	Live birth	Miscarriage
Age	0.907 (0.873–0.943)***	0.900 (0.866–0.937)***	0.870 (0.833–0.908)***	0.876 (0.839–0.914)***	1.118 (1.049–1.192)***
BMI	0.984 (0.946–1.023)	0.979 (0.939–1.20)	0.990 (0.946–1.037)	0.99 (0.945–1.036)	0.991 (0.929–1.056)
AMH	0.997 (0.943–1.054)	1.000 (0.946–1.057)	0.997 (0.940–1.057)	1.008 (0.951–1.069)	0.989 (0.904–1.081)
Basal FSH	0.955 (0.905–1.009)	0.978 (0.925–1.034)	0.989 (0.930–1.051)	0.998 (0.939–1.061)	0.967 (0.875–1.068)
Basal LH	1.046 (0.981–1.115)	1.035 (0.976–1.097)	1.056 (0.992–1.123)	1.035 (0.975–1.099)	0.972 (0.867–1.090)
Basal E2	0.999 (0.993–1.005)	1.002 (0.996–1.009)	0.999 (0.992–1.006)	1.000 (0.993–1.007)	1.005 (0.994–1.015)
Endometrial thickness	1.012 (0.931–1.100)	1.008 (0.927–1.096)	0.986 (0.901–1.079)	0.987 (0.902–1.081)	1.053 (0.927–1.196)
Morphological evaluation of blastocysts					
Blastocyst expansion	1.229 (1.001–1.511)*	1.336 (1.077–1.658)**	1.157 (0.913–1.465)	1.141 (0.900–1.447)	1.271 (0.895–1.804)
ICM	1.278 (0.92–1.776)	1.210 (0.862–1.700)	1.176 (0.809–1.710)	1.238 (0.849–1.804)	0.911 (0.523–1.587)
TE	1.174 (0.842–1.636)	1.333 (0.944–1.881)	1.339 (0.913–1.965)	1.242 (0.845–1.825)	0.986 (0.555–1.752)
Combined P4 dose–serum group	1.266 (1.117–1.434)***	1.412 (1.241–1.608)***	1.393 (1.209–1.605)***	1.349 (1.171–1.555)***	0.965 (0.780–1.193)

Abbreviations: AMH, anti‐Müllerian hormone; BMI, body mass index; CI, confidence interval; E2, estradiol; FSH, follicle‐stimulating hormone; GQB, good‐quality blastocyst; ICM, inner cell mass; LH, luteinising hormone; P4, progesterone; TE, trophectoderm.

* *p* < 0.05.

** *p* < 0.01.

*** *p* < 0.001.

### 3.3 Sensitivity Analysis

In addition, a sensitivity analysis was performed including only the first FET cycle per patient to account for potential within‐patient clustering. A total of 594 first FET cycles were analyzed, including 272 cycles in the P(90) group, 114 cycles in the P(300) group, and 208 cycles in the P(800) group. The ART outcomes observed in this sensitivity analysis were largely consistent with those of the main analysis including all cycles. These results are presented in Figure S2.

## 4 Discussion

The results of this study reveal that the daily dose of micronized vaginal P4 and serum P4 levels on the day of ET in HRT‐FET cycles positively influence ART outcomes, including implantation, clinical pregnancy, ongoing pregnancy, and live birth rates. The statistical cutoff value of serum P4 for predicting clinical pregnancy on the day of ET was 13.1 ng/mL, indicating that serum P4 levels may aid in predicting ART outcomes in HRT‐FET cycles using micronized vaginal P4.

This study demonstrates that increasing the daily dose of micronized vaginal P4 during HRT‐FET cycles improves ART outcomes. A comparison among the P(90), P(300), and P(800) groups showed a trend toward higher implantation, clinical pregnancy, ongoing pregnancy, and live birth rates with increasing daily doses of P4, with a statistically significant difference observed between the P(90) and P(800) groups. Similar findings have been previously reported: Enatsu et al. showed that ART outcomes were significantly better in the group receiving 1200 mg/day than in that receiving 900 mg/day of the same micronized vaginal P4, whereas Alsbjerg et al. demonstrated improved outcomes with 180 mg/day compared with 90 mg/day [11, 12]. These findings are consistent with the present results and support the conclusion that the daily dose of micronized vaginal P4 has a positive effect on ART outcomes. In contrast, Shiba et al. [10], in a randomized trial comparing different vaginal progesterone formulations with different dosing regimens, reported no significant differences in reproductive outcomes among the groups. Our findings therefore differ from those reported by Shiba et al. [10]. This discrepancy may be attributable to differences in study design and patient populations. In particular, Shiba et al. included both cleavage‐stage embryos and blastocysts and did not restrict the analysis to good‐quality blastocyst transfers [10], whereas our analysis focused on single good‐quality blastocyst transfers, which may reduce heterogeneity related to embryo stage and quality. In addition, differences in sample size and covariate adjustment may influence the precision of effect estimates and the consistency of observed associations in multifactorial outcomes such as ART success. Therefore, these findings should be interpreted within their respective clinical and methodological contexts. From a clinical perspective, the present results may lend support to considering a higher daily vaginal progesterone dose, particularly in patients in whom adequate serum P4 levels are not achieved with lower‐dose regimens. However, because this study was retrospective and observational, these findings should not be interpreted as establishing a uniform high‐dose strategy for all patients, and prospective studies are warranted to determine the optimal dosing approach.

In this study, increasing the daily dose of micronized vaginal P4 for luteal phase support during HRT‐FET cycles was associated with higher serum P4 levels on the day of ET. These serum P4 levels on the day of ET increased with higher daily doses, with statistically significant differences observed between P(90) and P(300), as well as between P(90) and P(800). Previous studies comparing daily doses of 200 and 300 mg/day of the same micronized vaginal P4 reported higher serum P4 levels in the high‐dose group [15], consistent with the findings of the present study. However, a previous study has reported results inconsistent with these findings [11]. These findings, consistent with earlier reports, suggest that higher daily doses of micronized vaginal P4 are associated with elevated serum P4 levels on the day of ET. Inconsistent results reported in prior studies may be attributed to differences in the timing of serum P4 measurement and differences in patient populations or study protocols.

Furthermore, in the present study, the optimal cutoff value of the serum P4 level on the day of ET for predicting clinical pregnancy was 13.1 ng/mL. ART outcomes were significantly higher in the group that met this threshold than in the group that did not, suggesting that serum P4 levels on the day of ET influenced ART outcomes in HRT‐FET cycles where micronized vaginal P4 was used for luteal phase support. Previous studies have frequently reported that higher serum P4 levels improve ART outcomes in HRT‐FET cycles where intramuscular P4 preparations were utilized [16, 17, 18]; nonetheless, the results of more recent studies, consistent with the current findings, have shown similar trends in cycles where micronized vaginal P4 were used. Initially, when micronized vaginal P4 were introduced, they were regarded as highly effective because of the first uterine pass effect, facilitating efficient delivery of P4 to the target organ: the endometrium. Therefore, serum P4 monitoring during HRT‐FET cycles using only micronized vaginal P4 is deemed unnecessary [9, 19]. In interpreting the discrepant conclusions across studies, the discrepant conclusions between our study and the prior reports by Shiba et al. and Fujiwara et al. may be partly attributable to differences in study populations and analytical approaches. In particular, variations in embryo‐related inclusion criteria (e.g., embryo stage and/or embryo quality), baseline patient characteristics, and covariate adjustment strategies may influence the magnitude and consistency of the association observed between serum P4 levels and reproductive outcomes. Differences in outcome definitions and follow‐up schedules may also contribute to variability across studies. Conversely, it has been increasingly recognized in recent studies that monitoring serum P4 levels is important, even in such cycles [20, 21, 22]. A plausible explanation is that P4 contributes to implantation and placentation and plays a critical role in maternal immune modulation during pregnancy, promoting an immunotolerant state conducive to successful gestation [21]. This immunomodulatory effect is believed to be dependent on the serum P4 concentration and may not be sufficiently compensated for by local endometrial P4 levels alone. Reports from a meta‐analysis investigating serum P4 levels during the luteal phase of ART showed that the optimal serum P4 concentration on the day of ET in HRT‐FET cycles using micronized vaginal [20] P4 ranged from 8 to > 15 ng/mL—a range that closely aligns with the cutoff value identified in the present study. Labarta et al. reported a serum P4 cutoff value of 9.2 ng/mL on the day of ET to improve ongoing pregnancy rates [23]. In a subsequent study, they demonstrated that applying individualized luteal phase support (iLPS)—in the form of additional subcutaneous P4 administration—for patients with serum P4 levels of < 9.2 ng/mL resulted in live birth rates comparable to those in patients with serum P4 levels of ≥ 9.2 ng/mL [24]. Similarly, the effectiveness of iLPS has been documented in several other studies involving the administration of additional subcutaneous or intramuscular P4 or oral dydrogesterone in cases where serum P4 levels on the day of ET were below the suggested cutoff [25, 26, 27]. These findings suggest that iLPS may be beneficial in cases with low serum P4 levels, regardless of the dosage of micronized vaginal P4 used, highlighting the need for further investigation into this approach.

In this study, the four groups were compared using daily P4 dosage and serum P4 levels on the day of ET. The results showed that the LL (< 300 mg, below the cutoff) group had significantly lower implantation and clinical pregnancy rates than the HL (≥ 300 mg, below the cutoff) and HH (≥ 300 mg, above the cutoff) groups. Moreover, the HH group exhibited significantly higher ongoing pregnancy and live birth rates than the LL and LH (< 300 mg, above the cutoff) groups. Although the LH group (low dosage but high serum P4 level) showed relatively high implantation and clinical pregnancy rates, its miscarriage rate was as high as 46.8%, resulting in ongoing pregnancy and live birth rates that were nearly equivalent to those of the LL group. Similarly, the HL group (high dosage but low serum P4 level) demonstrated relatively favorable implantation and clinical pregnancy rates, but its miscarriage rate was 38.5%, and its subsequent ongoing pregnancy and live birth rates were lower than those observed in the HH group. These findings suggest that serum P4 levels and daily P4 dosages play important roles in ART outcomes. Logistic regression analysis further demonstrated that the Combined P4 dose–serum group was an independent predictor positively influencing implantation, clinical pregnancy, ongoing pregnancy, and live birth, with adjusted odds ratios ranging from 1.226 to 1.412, thereby reinforcing these results. Additionally, in all dosage categories, groups with higher serum P4 levels (LH vs. LL and HH vs. HL) consistently achieved better ART outcomes than groups without. These results support the importance of monitoring serum P4 levels, as they demonstrate that serum P4 concentration is a factor influencing ART outcomes even in HRT‐FET cycles using micronized vaginal P4.

Although the present study was conducted at a single center, which may limit the generalizability of our findings, a major strength of this study is that all cycles were managed under a standardized protocol for luteal phase support using micronized vaginal P4. This uniform clinical practice reduced variability in treatment and measurement conditions, thereby enabling a consistent comparative analysis of serum P4 levels and ART outcomes among different vaginal progesterone formulations. The daily administration schedules for each micronized vaginal P4 used in this study were as follows: P(90) involved once‐daily administration of OneCrinone; P(300) involved three‐times‐daily administration of Lutinus; and P(800) involved twice‐daily administration of Luteum. On the day of ET, serum P4 levels were measured 6 h after administering OneCrinone and Luteum, and shortly after administration of Lutinus. According to the prescribing information, Lutinus and Luteum reach stable plasma concentrations by day 5 of administration [28, 29], while OneCrinone reaches peak plasma concentration approximately 6 h post‐administration [30]. By clearly defining the administration schedules and measurement timing, and by referencing the pharmacokinetic profiles of each formulation, this study ensures that the serum P4 levels accurately reflect the pharmacological efficacy of each micronized vaginal P4, strengthening the reliability and interpretability of the comparative analysis of ART outcomes among the different formulations. In addition, the present study evaluated progesterone supplementation by jointly considering the administered dose and achieved serum progesterone levels, providing a clinically intuitive framework to interpret ART outcomes in HRT‐FET cycles. However, a limitation of this study was its retrospective design, which precluded the standardization of micronized vaginal P4. Because each formulation differs in its physical characteristics and frequency of daily administration, these factors may influence P4 absorption and serum levels, making it challenging to evaluate the effect of the P4 dose alone [28, 29, 30]. In addition, the serum P4 cutoff value and dose‐based findings identified in the present study should be interpreted with caution. Although a cutoff of 13.1 ng/mL on the day of ET was associated with clinical pregnancy, its discriminative ability was limited, as reflected by the low ROC‐AUC. Likewise, although higher daily doses and the combined dose–serum P4 classification (LL/LH/HL/HH) were associated with differences in ART outcomes, these observations should not be interpreted as defining definitive clinical targets or decision thresholds. Given the retrospective observational design and the multifactorial nature of ART outcomes, the present findings—including the proposed cutoff, daily dose comparisons, and LL/LH/HL/HH classification—should be regarded as exploratory and hypothesis‐generating. Further prospective or interventional studies are warranted to validate clinically meaningful serum P4 thresholds and optimal dosing strategies, and to determine whether individualized luteal phase support based on serum P4 monitoring can improve reproductive outcomes.

In conclusion, our findings showed that in HRT‐FET cycles, the daily dose of micronized vaginal P4 positively influenced serum P4 levels and ART outcomes. Furthermore, serum P4 levels can serve as predictive markers for ART outcomes, even in HRT‐FET cycles where micronized vaginal P4 is used for luteal phase support. These findings suggest that the individualized selection of micronized vaginal P4 based on serum P4 levels may be important. Future investigations are needed to assess the usefulness of iLPS in cases where the serum P4 levels do not meet the cutoff value on the day of ET in HRT‐FET cycles utilizing micronized vaginal P4.

## Disclosure

Human Rights Statements and Informed Consent/Approval by Ethics Committee: The study was conducted in accordance with the ethical standards set forth in the 1964 Declaration of Helsinki and its subsequent amendments or comparable ethical standards. The requirement for written informed consent was waived because information regarding the study was provided on the hospital website in an opt‐out format, allowing potential research participants to decline participation.

## Ethics Statement

This study was approved by the Ethics Committee of the Toho University Omori Medical Center (approval no. M22239). Information about the study was publicly available on the hospital's website, allowing potential participants to opt out. The study was conducted with due consideration to ensure that non‐participation would not result in any disadvantages for the participants, with particular attention to the fact that all data were obtained retrospectively from existing medical records without any influence on clinical care, additional interventions, or costs.

## Conflicts of Interest

The authors declare no conflicts of interest.

## Supporting information


**FIGURE S1:** The calendar‐year distribution of micronized vaginal progesterone formulations used during the study period. The proportions of P(90), P(300), and P(800) administered in each calendar year are shown. P(90), OneCrinone 90 mg/day; P(300), Lutinus 300 mg/day; P(800), Luteum 800 mg/day.

**FIGURE S2:** Sensitivity analysis of reproductive outcomes including only the first frozen embryo transfer (FET) cycle per patient. A total of 594 first FET cycles were analyzed: P(90), *n* = 272; P(300), *n* = 114; P(800), *n* = 208. ART = assisted reproductive technology, P4 = progesterone, ET = embryo transfer. *p < 0.05; N.S, not significant.

**TABLE S1:** Patient characteristics of the four groups classified according to the daily dose of micronized vaginal progesterone and serum progesterone level.

## Data Availability

The data that support the findings of this study are available from the corresponding author upon reasonable request.
