# Supplementary material for: Micronized Vaginal Progesterone Dose and Serum Progesterone Thresholds Determine Reproductive Outcomes in Frozen–Thawed Embryo Transfer With Hormone Replacement Therapy
Source: Reprod Med Biol. 2026 Mar 12;25(1):e70039. doi: 10.1002/rmb2.70039 (PMC13045485; doi:10.1002/rmb2.70039)
Supplement: Supplementary file 3 — TABLE S1: Patient characteristics of the four groups classified according to the daily dose of micronized vaginal progesterone and serum progesterone level. [file RMB2-25-e70039-s002.docx]

Supplemental Table 1

Patient characteristics of the four groups classified according to the daily dose of micronized vaginal progesterone and serum progesterone level

|  | LL n=363 | LH n=157 | HL n=282 | HH n=335 | P value |
| --- | --- | --- | --- | --- | --- |
| Female age (years) | 37(24-48)^a^ | 38(27-48)^b^ | 38(22-45)^b^ | 38(25-45)^b^ | 0.002 |
| BMI（㎏/㎡） | 22.3±3.7^a^ | 21.8±3.9^b^ | 22.6±3.9^a^ | 22.4±3.7^a^ | 0.03 |
| AMH(ng/mL) | 3.3±2.7 | 3.0±2.8 | 3.1±2.6 | 3.5±3.1 | 0.354 |
| Baseline FSH (mIU/mL) | 7.2±2.8^a^ | 8.3±3.2^b^ | 7.3±3.1^ac^ | 7.7±3.5^c^ | <0.001 |
| Baseline LH (mIU/mL) | 4.9±3.4^ac^ | 5.5±2.6b | 4.4±2.4^a^ | 5.0±2.5^c^ | <0.001 |
| Baseline E2 (pg/mL) | 37.0±25.5^ac^ | 45.3±22.1^b^ | 32.8±22.9^a^ | 37.4±23.3^c^ | <0.001 |
| Endometrial Thickness on ET Scheduling Day（mm） | 9.7(7.0-15.9) | 10.1(6.4-17.3) | 9.9(6.2-18.7) | 10.0(7.0-19.0) | 0.11 |
| Serum E2 level on ET Day(pg/mL) | 292±172^a^ | 296±208^a^ | 248±152^b^ | 301±192^a^ | 0.001 |
| Serum P4 level on ET Day(ng/mL) | 9.2±2.4^a^ | 17.0±3.9^b^ | 9.9±2.4^c^ | 18.7±5.8^b^ | <0.001 |
| Serum P4 level on Pregnancy test Day(ng/mL) | 9.4±11.5a | 12.6±6.6b | 11.8±7.7b | 17.3±9.3c | <0.001 |

For continuous variables, data are expressed as the median (range: minimum–maximum) or as the mean ± standard deviation. Superscript letters indicate statistical comparison: values sharing the same letter are not significantly different, whereas values with different letters differ significantly.

**Abbreviations:** BMI, body mass index ;AMH, anti-Müllerian hormone; FSH, follicle-stimulating hormone; LH, luteinizing hormone; ET, embryo transfer
